# Multimodal Proteomics Reveals Dysregulated Secretion and ECM Remodelling in Schizophrenia Patient iPSC-Derived Astrocytes

**DOI:** 10.3390/cells15121052

**Published:** 2026-06-09

**Authors:** Wei-Ping Li, Karen E. Laupman, Stephanie D. Beekhuis-Hoekstra, Evangelia Thanou, Remco V. Klaassen, Patrick F. Sullivan, Danielle Posthuma, August B. Smit, Frank Koopmans, Vivi M. Heine

**Affiliations:** 1 Department of Complex Trait Genetics, Center for Neurogenomics and Cognitive Research, Vrije Universiteit Amsterdam, 1081 HZ Amsterdam, The Netherlands; 2 Department of Molecular and Cellular Neurobiology, Center for Neurogenomics and Cognitive Research, Vrije Universiteit Amsterdam, 1081 HZ Amsterdam, The Netherlandsfrank.koopmans@vu.nl (F.K.); 3 Department of Medical Epidemiology and Biostatistics, Karolinska Institutet, 171 77 Stockholm, Sweden; 4 Department of Genetics, University of North Carolina, Chapel Hill, NC 27599-7264, USA; 5 Department of Child and Adolescent Psychiatry, Amsterdam Neuroscience, Vrije Universiteit Medical Center, 1081 HV Amsterdam, The Netherlands

**Keywords:** schizophrenia, astrocytes, protein secretion, iPSC, proteomics

## Abstract

Astrocytes are increasingly implicated in the pathophysiology of schizophrenia (SCZ), yet how astrocytic dysfunction contributes to disease-relevant neuronal abnormalities remains unclear. Here, we used mass spectrometry–based proteomics to profile lysates (proteome) and secreted proteins (secretome) from iPSC-derived astrocytes originating from 9 SCZ patients and 8 healthy controls. Compartment-specific analyses showed that lysates were enriched for mitochondrial and nuclear pathways, whereas astrocyte-conditioned media (ACM) were enriched for extracellular matrix (ECM) and vesicle-associated proteins. Differential expression analysis revealed minimal overlap between dysregulated proteins in lysates and ACM, suggesting modality-specific effects of SCZ-associated donor background. Interestingly, ECM proteins and key secreted cues involved in synaptic development, including MFGE8 and SEMA3C, were selectively reduced in SCZ ACM, whereas RNA-processing proteins were aberrantly increased. This is in line with previously reported microRNA enrichment in extracellular vesicles (EV) derived from SCZ patients. Gene set analyses further identified the alteration in secretion and nuclear processes as well as the potential involvement of autophagy-dependent release mechanism in SCZ astrocytes. Together, these findings suggest disrupted astrocytic protein homeostasis and extracellular signalling in SCZ iPSC-derived astrocytes, providing mechanistic insight into astrocyte-mediated contributions to synaptic and circuit deficits in the disorder.

## 1. Introduction

Schizophrenia (SCZ) is a debilitating psychiatric disorder with an estimated worldwide prevalence of 0.33–0.75% [1,2]. Core clinical features of SCZ include positive symptoms (e.g., delusions, hallucinations and disorganised thoughts), negative symptoms (such as impaired motivation, reduced expression of emotions and social withdrawal), and cognitive impairment (i.e., dysfunction of attention, intellectual ability and memory) [3,4]. Although the first episode of psychosis commonly occurs in late adolescence or early adulthood, SCZ is widely considered as a neurodevelopmental disorder [4,5]. Despite extensive research, the molecular mechanisms underlying SCZ remain largely elusive.

While neurons have long been the focus of psychiatric research, increasing evidence implicates astrocytes in SCZ pathophysiology. For example, a recent anatomical study reported a significant decrease in astrocyte density in the subgenual cingulate cortex and anterior corpus callosum in individuals with SCZ [6]. Similarly, mRNA levels of several astrocyte markers, including S100β, AQP4 and EAAT2, were significantly reduced in the deep layers of the anterior cingulate gyrus in SCZ brain tissue [7]. In addition, many transcriptomics studies reported the up-regulation of astrocyte-specific genes [8], astrocyte marker gene profiles [9], and astrocyte-associated gene co-expression modules [10] in cortical samples from individuals with SCZ. Furthermore, genes differentially spliced in SCZ cortex were found enriched in astrocytes [11]. Although these studies may not fully agree on changes in astrocyte numbers, they collectively point to astrocyte abnormalities in SCZ.

Synaptic loss is a well-characterised microscale phenotype of SCZ revealed by neuromorphological analyses [12,13] and by quantification of synaptic mRNAs and proteins [14,15,16,17,18] in postmortem brain tissue as well as in vivo positron emission tomography (PET) scans [19,20]. Astrocytes are known to modulate the formation, maturation and elimination of synapses via their secreted signals [21]. For example, astrocytes secrete thrombospondins [22] and hevin [23] to promote synaptogenesis in vitro and in vivo. In the retinogeniculate system, secreted factors from astrocytes up-regulate the expression of complement component 1q (C1q) in retinal ganglion cells (RGCs), potentially triggering the classical complement cascade and microglia-mediated synapse elimination [21,24]. Taken together, these findings suggest that aberrant astrocyte secretion may contribute to synaptic deficits in SCZ.

To further investigate the role of astrocytes in SCZ pathophysiology [25], we characterised protein expression and secretion in astrocytes derived from individuals with schizophrenia by profiling the proteome and secretome of induced pluripotent stem cell (iPSC)-derived astrocytes (8 control and 9 SCZ iPSC lines) using mass spectrometry. Our analyses reveal the differential impacts of ‘SCZ-associated donor background’—reflecting the polygenic nature of the disorder—on the astrocytic proteome and secretome and provide insight into the strengths and limitations of these complementary proteomic approaches.

## 2. Materials and Methods

### 2.1. Patient Selection and iPSC Generation

Fibroblasts were reprogrammed using a previously described protocol [26]. All iPSC lines used for experiments were cultured on Vitronectin™-coated surfaces (Stem Cell Technologies, Vancouver, BC, Canada, 7180) in TeSR™-E8™ medium (Stem Cell Technologies, 5940) with 10 µM ROCK inhibitor (RI; Y-27632). iPSC culturing and maintenance was performed using a previously described protocol [26,27]. In this study, 9 SCZ lines and 8 control lines are used, which are all registered on hPSCreg.eu.

### 2.2. Astrocyte Differentiation and Maintenance

Astrocytes were differentiated as previously described [28] with minor adjustments. To induce astrocyte differentiation the cells were switched to astrocyte medium (with the provided supplements FBS, AGS and P/S, ScienCell, Carlsbad, CA, USA,1801) around day 45. During the maturation phase and to stop proliferation, 2 days after splitting around day 75 of the differentiation, FBS was omitted from the medium 2 days after the last passage. The medium was refreshed two to three times a week, decreasing with maturity, and cells were passaged with 10 µM RI when full confluency was reached, ranging from 7 days up to 14 days approaching the end of the protocol. QC of the astrocyte differentiation was performed between day 50 and 60 by immunocytochemistry using the markers GFAP, CD44, SOX9, Nestin, and ID3. Astrocytes progenitors were frozen at various time points, but astrocyte analysis was performed after at least 60 days of continuous culturing.

### 2.3. ACM and Astrocyte Collection

For the collection of the ACM, the astrocytes were plated at day 81 in 12-well plates at a density of 15,000/cm^2^. At day 82, the medium was completely switched to Neurobasal after two washing steps with PBS. At day 90, the culture medium was collected and spun down at 5000× *g*. The supernatant was transferred to another tube, snap-frozen and stored at −80 °C. The astrocytes were plated at a density of 15,000/cm^2^ at day 96 and collected 4 days later with a cell scraper (Greiner, Frickenhausen, Germany, 541070) at day 100, spun down at 13,000× *g* at RT in PBS. Dry cell pellets were snap-frozen.

### 2.4. MS Sample Preparation of Astrocytes

Astrocyte pellets were extracted and reduced in a Laemmli sample buffer, containing 5 mM tris(2-carboxyethyl)phosphine (TCEP), by incubation at 55 °C for 15 min in a thermomixer (Eppendorf SE, Hamburg, Germany) set to 1250 RPM. Free sulfhydryl groups were alkylated by incubation with 20 mM methyl methanethiosulfonate (MMTS) for 15 min at RT. Next, the lysates were cleared of insoluble debris by centrifugation at 15,000× *g* for 1 min at RT, loaded onto a 10% SurePAGE polyacrylamide gel (Genscript, Piscataway, NJ, USA) and resolved for 1 cm. The gels were fixed overnight and stained with colloidal Coomassie Blue G-250. Sample lanes were cut into 1 mm^3^ cubes, transferred to a MultiScreen HV 96-well filter plate (Merck Millipore, Darmstadt, Germany) and destained until clear using repeated applications of 50 mm NH_4_HCO_3_ in 50% acetonitrile. After dehydration with 100% acetonitrile, each well was supplemented with 0.4 µg MS grade Trypsin/Lys-C (Promega) in 50 mM NH_4_HCO_3_ and tryptic digestion was performed overnight at 37 °C within a humidified incubator. Tryptic peptides were extracted and pooled by two incubations with 0.1% TFA in 50% acetonitrile, dried by SpeedVac (Concentrator plus from Eppendorf, Hamburg, Germany), and stored at −80 °C.

### 2.5. MS Sample Preparation of Astrocyte Conditioned Medium

MS sample preparation of astrocyte-conditioned medium was performed following the DNA micro spin column suspension trapping (S-Trap) protocol [29], with some minor adaptations. The medium was mixed with S-trap lysis buffer up to a final concentration of 5% SDS, 100 mM Tris-HCl pH 8.0 and 2 mM tris(2-carboxyethyl)phosphine (TCEP), and incubated in a thermomixer for 30 min at 65 °C. Free sulfhydryl groups were alkylated by incubation with 8 mM methyl methanethiosulfonate (MMTS) for 5 min at RT. Next, the samples were acidified to a final concentration of 1.1% phosphoric acid (12% stock solution), mixed with six volumes of a binding/washing buffer (90% methanol and 100 mM Tris-HCl pH 8.0), and loaded onto a plasmid DNA micro column (HiPure from Magen Biotechnology Co., Guangzhou, China). The protein particulate was retained on the column upon centrifugation at 1400× *g* for 1 min and the columns were washed four times with binding/washing buffer. Columns were transferred to new LoBind tubes (Eppendorf, Hamburg, Germany), supplemented with 0.4 μg Trypsin/Lys-C (Promega, Madison, WI, USA) in 50 mM NH_4_HCO_3_ and incubated overnight at 37 °C in a humidified incubator. Tryptic peptides were eluted and pooled by subsequent addition of 50 mM NH_4_HCO_3_, 0.1% formic acid and 0.1% formic acid in acetonitrile. Collected peptides were dried by SpeedVac and stored at −80 °C.

### 2.6. LC-MS Analysis

Each sample of tryptic digest was redissolved in 0.1% formic acid and the peptide concentration was determined by tryptophan-fluorescence assay [30]; 75 ng of peptide was loaded onto an Evotip Pure (Evosep, Odense, Denmark). Peptide samples were separated by standardised 30 samples per day method on the Evosep One liquid chromatography system, using a 15 cm × 150 μm reverse-phase column packed with 1.5 µm C_18_-beads (EV1137 from Evosep) connected to a 20 µm ID ZDV emitter (Bruker Daltonics, Billerica, MA, USA).

Peptides were electro-sprayed into the timsTOF Pro 2 mass spectrometer (Bruker Daltonics) equipped with CaptiveSpray source and measured with the following settings: Scan range 100–1700 *m*/*z*, ion mobility 0.65 to 1.5 Vs/cm^2^, ramp time 100 ms, accumulation time 100 ms, and collision energy decreasing linearly with inverse ion mobility from 59 eV at 1.6 Vs/cm^2^ to 20 eV at 0.6 Vs/cm^2^. Operating in dia-PASEF mode, each cycle took 1.59 s and consisted of 1 MS1 full scan and 14 dia-PASEF scans. Each dia-PASEF scan contained two isolation windows, in total covering 300–1301 *m*/*z* (1 Th window overlap) and ion mobility 0.65 to 1.50 Vs/cm^2^. Dia-PASEF window placement was optimised using the py-diAID tool v0.0.18 [31]. Ion mobility was auto-calibrated at the start of each sample (calibrant *m*/*z*, 1/K0: 622.029, 0.992 Vs/cm^2^; 922.010, 1.199 Vs/cm^2^; 1221.991, 1.393 Vs/cm^2^).

### 2.7. MS Downstream Analysis

DIA-PASEF raw data were processed with DIA-NN 1.8.1 [32,33]. An in silico spectral library was generated from the uniprot human proteome (SwissProt and TrEMBL, canonical and additional isoforms, release 2022-03) using Trypsin/P digestion and at most 1 missed cleavage. Fixed modification was set to beta-methylthiolation(C) and variable modifications were oxidation(M) and N-term M excision (at most 1 per peptide). Peptide length was set to 7–30, precursor charge range was set to 2–4, precursor *m*/*z* was limited to 280–1320, both MS1 and MS2 mass accuracy were set to 10 ppm, scan window was set to 8, double-pass-mode and match-between-runs were enabled. Protein identifiers (isoforms) were used for protein inference. All other settings were left as default.

MS-DAP 1.0.9 [34] was used for the downstream analyses of the DIA-NN results. Filtering and normalisation were applied to the respective samples per statistical contrast. Peptide-level filtering was configured to retain only peptides that were confidently identified in at least 75% of the samples per sample group. Peptide abundance values were normalised using the VSN algorithm, followed by protein-level mode-between normalisation (MS-DAP defaults).

### 2.8. Assessment of Protein Enrichment Across Modalities and Derivation of Enrichment Scores

The enrichment of proteins was assessed in Ctrl and SCZ samples independently. DIA-NN output ‘pg_matrix’ (containing normalised MaxLFQ quantities for protein groups) from lysate and ACM datasets were used as the input. To address the ambiguous protein groups, we aggregated the intensity of the proteins that share the identical leading gene symbol. Only the proteins that were detected in at least two samples in both modalities were used. Mode Within, Mode Between (MWMB) normalisation algorithm in MS-DAP package was then applied to make the samples from the lysate and ACM datasets comparable. Finally, the enrichment scores of the proteins were derived from the ratio of their mean intensity across the samples in the lysate dataset to their mean intensity in the ACM dataset on log_2_ scale, i.e., log_2_(mean intensity in lysates/mean intensity in ACM).

To prepare the enrichment scores for GO analyses using the GOAT algorithm, which uses the rank order of input genes to compute gene set enrichment, we replaced infinite enrichment scores with rank order-preserving scores. All proteins observed in lysates but not ACM, or vice versa, yield an infinite value for log_2_(mean intensity in lysates/mean intensity in ACM) and thus their enrichment scores are ties. We thus imputed enrichment scores to break these ties such that the order of respective mean abundance values in lysates or ACM was preserved. Because the GOAT algorithm operates on rank-transformed input gene scores, the rank order of imputed values is important while their absolute values are immaterial. Proteins only detected in lysates were first ordered based on their normalised abundance. The least abundant lysate-specific protein was then assigned a score equal to the maximum finite enrichment score (from the protein detected in both modalities) plus 1. The remaining proteins were assigned imputed scores in ascending order with an increment of 0.001. Similarly, proteins exclusively detected in ACM were first ordered based on their normalised abundance. The least abundant ACM-specific protein was assigned a score equal to the minimum finite enrichment score minus 1. The remaining proteins were then assigned imputed scores in descending order at intervals of 0.001.

**Figure 1:**
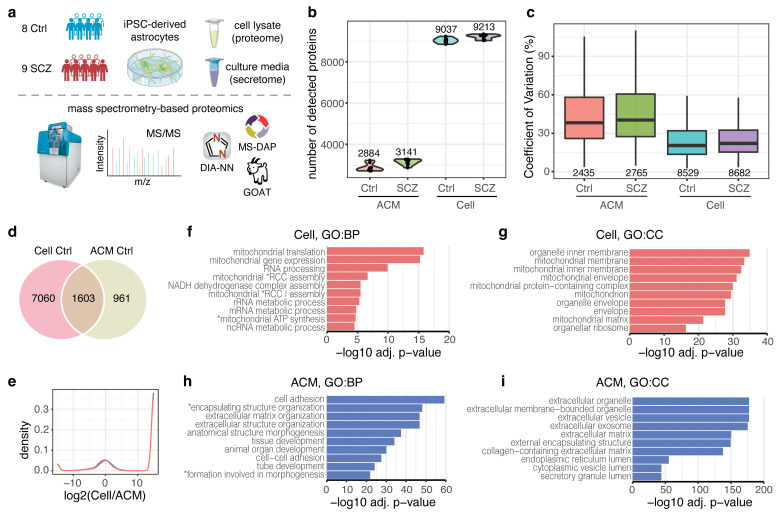
Proteomic analysis of iPSC-derived astrocyte cell lysates and ACM. (**a**) Overview of the experimental design: lysates and media of iPSC-derived astrocytes were interrogated using mass spectrometry. (**b**) Number of detected proteins in each experimental condition. The mean value is shown for each sample group. (**c**) Distributions of protein (abundance) variation in each experimental condition. Only proteins identified in at least 3 replicates (protein numbers are shown at the bottom) were used for visualisation. (**d**) Overlap of proteins consistently detected (≥75% samples/group) in control lysates and ACM. (**e**) Distribution of lysate/ACM enrichment ratios. Colour-coded lines represent ratios computed within control (blue) and SCZ (red) samples. (**f**,**g**) Top 10 biological process (BP) and cellular component (CC) terms that were enriched in control lysate samples as compared to ACM (Table S3). (**h**,**i**) Analogous to panels (**f**,**g**), but showing GO terms enriched in control ACM as compared to lysate samples (Table S3). Abbreviations in panels (**f**,**h**) (indicated by *): RCC, respiratory chain complex; mitochondrial ATP synthesis, proton motive force−driven mitochondrial ATP synthesis; encapsulating structure organisation, external encapsulating structure organisation; formation involved in morphogenesis, anatomical structure formation involved in morphogenesis.

**Figure 2:**
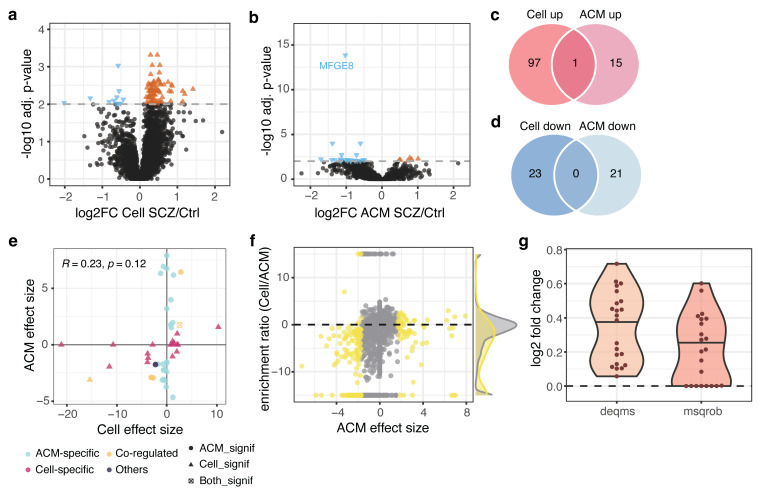
Investigating SCZ-associated dysregulation at the protein level. (**a**,**b**) Volcano plots from differential expression analyses (DEA) that compare control and SCZ conditions in lysate (**a**) and culture media (ACM) (**b**). (**c**,**d**) Overlap of proteins significantly up-regulated (**c**) and down-regulated (**d**) in SCZ lysates and ACM. This incorporates significant proteins from both DEA (**a**,**b**) and differential detection analyses. (**e**) Control versus SCZ effect sizes in lysates (*x*-axis) and ACM (*y*-axis) for 46 proteins that were tested in both modalities and significant (**c**,**d**) in either. Colours denote the classification of proteins strongly dysregulated in either or both (co-regulated) modalities (see Section 2.10.). Shapes indicate statistical significance. (**f**) Relationship between the control versus SCZ effect sizes in ACM (*x*-axis) and enrichment ratios (*y*-axis; control ratios in Figure 1e). The top 10% proteins with strongest (absolute) effect sizes in SCZ ACM are highlighted in yellow. (**g**) Histone proteins are enriched in SCZ lysates as compared to controls, suggesting an increased number of cells in the SCZ samples. Fold changes for each protein are shown (*y*-axis) for both the MSqRob and DEqMS statistical models (*x*-axis). Median fold changes: DEqMS—1.32; MSqRob—1.16.

For the clarity of visualisation in Figure 1e and Figure 2f and Figure S2f, we assigned +15 and −15 as the enrichment score for the proteins exclusively detected in lysates and ACM, respectively.

### 2.9. Gene Set Analysis Using GOAT

The GOAT R package v1.0 [35] was used to perform gene-set enrichment analyses with the GOAT algorithm using gene sets from the Gene Ontology database (downloaded on 1 January 2024).

For the functional enrichment in lysates and ACM within the context of Ctrl or SCZ, we first filtered the proteins that were detected in at least 75% of the samples in each condition (i.e., Ctrl_lysates, Ctrl_ACM, SCZ_lysates, and SCZ_ACM). Next, we identified the union of the unique (leading) gene symbols of the filtered proteins from lysates and ACM samples, and this step was carried out for Ctrl and SCZ samples independently. The protein enrichment scores with imputed values for lysate- and ACM-specific proteins (column ‘log2fc_goat’ in Table S2) were then joined to the two sets of proteins (lysate&ACM from Ctrl or SCZ) based on the genetic background, and we ended up with 7409 and 7543 unique gene symbols with associated enrichment scores from Ctrl and SCZ samples, respectively, as the input gene lists for the GOAT analyses. One-way test was applied using the enrichment scores as the ‘effectsize’: in the function test_genesets(), we set score_type = ‘effectsize_up’ to test for the enrichment in lysates, whilst score_type = ‘effectsize_down’ was set to test for the enrichment in ACM. The remaining settings remained as default. In short, we filtered the gene sets that contained at least 10 and at most 1500 genes that overlapped with our input gene lists. Two-step multiple testing correction was implemented: Bonferroni correction was first applied for the gene sets in three GO domains (column ‘source’ in Table S3) independently, and all the *p*-values were adjusted again using Bonferroni correction to account for the three independent tests for three GO domains. The significance threshold for the adjusted *p*-values was set to 0.05.

As for the case-control comparison of the lysates and ACM, dea+dd statistics from MS-DAP (Table S4) were directly used as the input gene lists (lysates: 7155 genes; ACM: 2098 genes) for the GOAT analyses. A two-way test was applied by setting score_type = ‘effectsize’ in the function test_genesets(). The settings for gene-set filtering, multiple testing correction, and significance threshold are the same as described above.

### 2.10. Classification of Modality-Specific Dysregulated Proteins

To identify the proteins that were specifically dysregulated in either lysates or ACM or co-regulated in both, we first joined MS-DAP dea+dd statistics from the two datasets using the column ‘gene’ (Human Entrez IDs). After filtering the proteins that were significant in either lysates or ACM, we first designated the proteins that (1) were significant in both modalities or (2) had absolute effect size ≥ 2 and shared the same direction of regulation in both modalities as ‘co-regulated’ proteins. Secondly, the proteins that were exclusively significant in ACM and had absolute effect size < 2 in lysates were labelled as ‘ACM-specific’ proteins. Thirdly, the proteins that were only significant in lysates and had absolute effect size < 2 in ACM were classified as ‘cell-specific’ proteins. Lastly, we assigned the proteins that did not meet any aforementioned criteria to the group ‘others.’

## 3. Results

### 3.1. Proteomic Analysis of iPSC-Derived Astrocytes

To investigate the dysregulation in astrocytes derived from individuals with schizophrenia in terms of gene expression and secretion, we differentiated iPSCs from eight healthy controls and nine SCZ donors into astrocytes, then collected cell lysates and the astrocyte-conditioned media (ACM) for proteomics analysis (Figure 1a, Table S1). All the samples were measured on a Bruker TimsTOF Pro2 mass spectrometer, and the data was processed with DIA-NN [32] and then subjected to downstream quality control and statistical analysis using MS-DAP [34]. On average, we identified 16,398 peptides from 2884 proteins in control ACM samples and 84,842 peptides from 9037 proteins in control lysate samples. Similarly, 18,287 peptides from 3141 proteins and 87,974 peptides from 9213 proteins were detected in SCZ ACM and lysate samples, respectively (Figure 1b and Figure S1a). Variation in protein abundance in the lysates was generally lower than in the ACM with a median coefficient of variation (CV) of 20.6% versus 38.2% in controls and 22.2% versus 40.3% in SCZ samples (Figure 1c and Figure S1b). Among consistently detected proteins (≥75% of samples), 8663 were identified in control lysates and 2564 in control ACM; 1603 overlapped (16.7% of 9624 unique proteins) (Figure 1d). In SCZ astrocytes, 8793 lysate proteins and 2836 ACM proteins shared an overlap of 1736 proteins (17.5% of 9893 unique proteins) (Figure S1c).

To assess the differences in protein abundance between lysate and ACM, we computed the log_2_ fold changes of protein intensities (abundance values) for the subset of 9624 proteins using the mean value across replicates in the respective control sample groups (Figure 1e, Table S2). Consistent with the Venn diagram distributions (Figure 1d), most proteins were strongly enriched (or exclusively detected) in either lysates or ACM. A highly similar distribution was obtained for lysate-vs-ACM ratios within SCZ samples, which suggested that the SCZ-associated donor background did not alter the general pattern of relative enrichment over compartments.

To characterise the biological processes underlying these differences, we performed Gene Ontology (GO) analysis using the gene ordinal association test (GOAT) [35] on the enrichment ratios (Figure 1f–i and Figure S1d–g, Table S3). In the control samples, the lysates were enriched for mitochondrial processes and components as well as RNA metabolic processes (Figure 1f,g), whereas ACM was enriched for GO terms related to extracellular matrix, vesicle and cell adhesion (Figure 1h,i). As expected, this same enrichment pattern was observed in both lysates and ACM when repeating this analysis on the samples from SCZ patients (Figure S1d–g).

### 3.2. SCZ-Associated Donor Background Differentially Affects Astrocytic Proteome and Secretome

Control versus SCZ differential expression analysis (DEA) using MSqRob [36] identified 88 dysregulated proteins in lysates and 28 in ACM (FDR < 0.01) (Figure 2a,b, Table S4). The distributions of (plain) log_2_ fold changes were centred around zero, reflecting that the data were normalised properly (Figure S2a). The lower number of significant ACM hits could be partially attributed to higher variation between secretome replicates (Figure 1c and Figure S1b). To capture low-abundance proteins in either experimental condition, we performed differential detection (DD) analysis. We identified 34 additional proteins in lysates and 10 proteins in ACM with strong control versus SCZ differences (Figure S2b,c).

Effect sizes from DEA and DD were then aggregated using MS-DAP [34] to enable a series of comparisons between SCZ astrocyte lysates and ACM. Strikingly, we identified only one protein in the overlap of significant proteins, an RNA-binding protein heterogeneous nuclear ribonucleoprotein R (HNRNPR), which was up-regulated in SCZ in both lysate and ACM samples (Figure 2c,d). In addition, correlations of protein effect sizes between lysates and ACM were modest (Pearson’s r = 0.25 for common proteins, Figure S2d; r = 0.23 for significant proteins, Figure 2e and Figure S2e). These results suggest that SCZ-associated alterations in the astrocytic proteome and secretome were only partially shared.

Based on the significance and effect direction, 46 dysregulated proteins were grouped into ACM-specific, lysate-specific, co-regulated proteins and ‘others’ categories (Figure 2e and Figure S2e). Notably, 23 proteins in the ACM-specific group likely arose from impaired secretion in diseased astrocytes rather than changes in intracellular protein expression. These proteins may reflect altered secretion dynamics in SCZ astrocytes, although contributions from technical variability in the ACM dataset cannot be excluded. Two strongly down-regulated secreted proteins in this group are well-known: milk fat globule-epidermal growth factor 8 (MFGE8), which regulates neurogenesis in rodent hippocampus [37,38] and is associated with synapse removal in Alzheimer’s disease [39,40], and Semaphorin 3C (SEMA3C), which plays a role in axon guidance [41,42].

Amongst 17 lysate-specific dysregulated proteins, extracellular matrix proteins (COL12A1, COL14A1 and MXRA5) as well as cytoskeletal and motor proteins (KRT16, DNM2, CAP1, KIF20B and MYH9) showed the strongest disease effect sizes in the lysates. Five proteins (ANGPT1, HNRNPR, TAGLN, MCAM and SDK1) exhibiting strong effect sizes in both modalities (proteome and secretome) were classified as co-regulated proteins. Lastly, since MYDGF was significant in the DEA of ACM but showed large effect size in the lysates, it was assigned to the ‘others’ group. This classification provides a useful means of identifying proteins exclusively affected in each modality and emphasises the dysfunctional secretion in SCZ astrocytes.

Moreover, comparing the relative enrichment between lysate and ACM (using ratios from Figure 1e) with DEA effect sizes from ACM dataset showed that proteins with top 10% effect sizes were also relatively enriched in the ACM (Figure 2f). On the other hand, proteins with top 10% effect sizes from the lysate dataset were less enriched in the cell lysates in comparison with the other proteins (Figure S2f). The concurrence evinces that there existed a population of proteins that were affected by the SCZ-associated donor background and best studied in ACM (as opposed to lysates). These findings suggest that the most strongly altered proteins in SCZ astrocytes are secreted factors, underscoring the relevance of studying the astrocytic secretome in psychiatric diseases such as schizophrenia.

Previous anatomical studies revealed the reduced somal size of layer III pyramidal neurons in the prefrontal cortex [43,44] and auditory association cortex [45] of SCZ patients. Cell size differences could confound interpretation because equal protein input per sample may represent different cell numbers. To test this, we inspected control versus SCZ fold changes of histones in cell lysates, as their abundance scales with DNA content and hence cell number [46]. The fold changes of an assortment of histone proteins retrieved from HistoneDB2.0 [47] were consistently greater or equal to zero (median fold changes: DEqMS = 1.32, MSqRob = 1.16; Figure 2g), but none reached statistical significance. While increased histone copy numbers per equal-protein input suggest higher astrocyte counts, their lack of significant regulation implies this does not impact the interpretation of significant hits (Figure 2a).

For the ACM dataset, a slight increase of histones in SCZ samples was again observed (median fold changes: DEqMS = 1.48, MSqRob = 1.00; Figure S2g), yet none of them was statistically significant. Unlike the case of cell lysates, the histone proteins in ACM might come from the cell debris. Slight histone elevation in ACM suggests greater vulnerability and debris leakage in SCZ cells, though this does not account for major findings.

### 3.3. Gene Set Analysis Implicates Altered Release of Extracellular Vesicles Containing RNAs by SCZ Astrocytes

**Figure 3:**
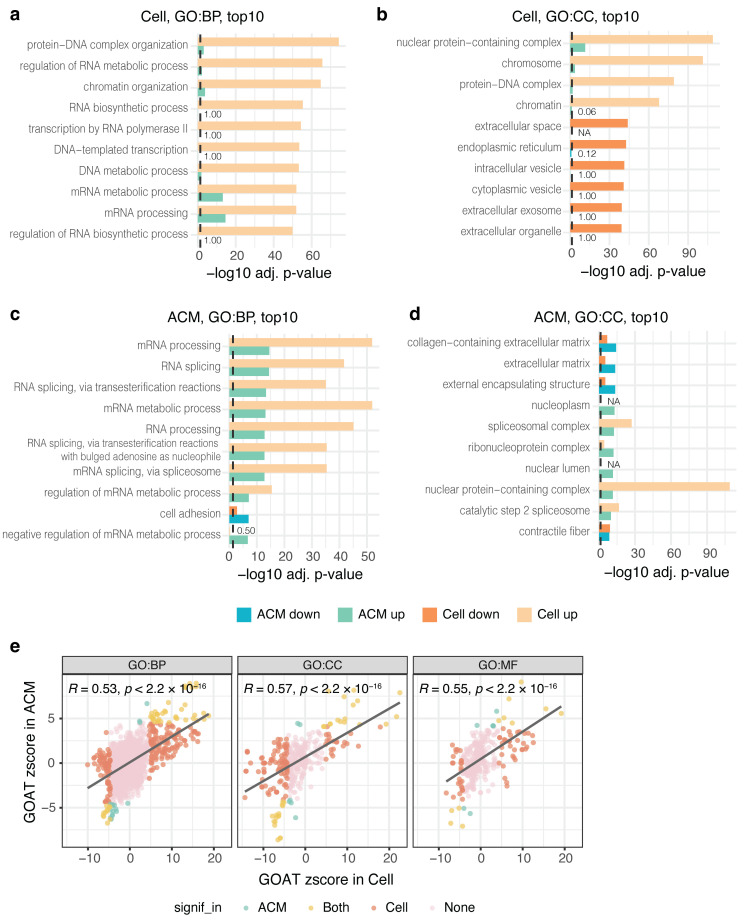
Investigating SCZ-associated dysregulation at the gene-set level. (**a**,**b**) Top 10 biological process (BP; (**a**)) and cellular component (CC; (**b**)) terms identified by gene set analysis of lysate samples. (**c**,**d**) Top 10 BP (**c**) and CC (**d**) terms identified by gene set analysis of ACM samples. The dataset from which the significance (*p*-value) was derived is colour-coded in teal (ACM) and orange (lysates). Lighter and darker colours denote up- and down-regulation, respectively. (**e**) Correlation of GOAT z-scores between lysate (*x*-axis) and ACM (*y*-axis) datasets. 2446 BP, 363 CC, and 348 MF terms, which were included in the gene set analyses of the two datasets, are used in the correlation analysis here. Pearson’s correlations are shown. GO terms are colour-coded by the dataset(s) in which they were statistically significant.

To understand the cell functions that were perturbed in the SCZ astrocytes, we performed gene set analysis of our cell lysate and ACM datasets, which captured proteome and secretome, respectively, using GOAT [35]. Numerous biological processes were significantly altered in the SCZ proteome (Table S5). Nuclear processes, such as DNA/RNA metabolic process and chromatin organisation, were the most significant terms, followed by secretion (e.g., protein secretion, vesicle-mediated transport, and vesicle organisation), cell cycle, actin filament organisation, cell adhesion, and translation (Figure 3a and Figure S3a). Fewer significant terms were identified for the secretome (Table S5), partially due to limited number of proteins detected (Figure 1b). Amongst the significant terms were (m)RNA processing and splicing, cell adhesion, extracellular matrix (ECM) organisation, and actin cytoskeleton organisation (Figure 3c and Figure S3b).

Since similar pathways were found to be dysregulated in the SCZ proteome and secretome, i.e., up-regulation of nuclear processes and down-regulation of ECM and secretion (Figure 3a–d and Figure S3c,d), we investigated the correlation of the effect sizes (z-scores) of all 3157 GO terms that were tested against both datasets and found the three GO domains (number of tested terms: Biological Process (BP) = 2446, Cellular Component (CC) = 363, Molecular Function (MF) = 348) were all substantial (Pearson’s r: BP = 0.53, CC = 0.57, MF = 0.55; Figure 3e), indicating overall similar trends of SCZ effects on the two modalities (proteome and secretome).

Next, we zoomed in on the top 10 significant terms identified in each GO domain. In BP, DNA/RNA metabolic process and chromatin organisation topped the list from cell lysates (Figure 3a). Nine out of the top ten significant terms derived from the ACM concerned up-regulated RNA processing and splicing, whilst cell adhesion molecules were found down-regulated in both modalities (Figure 3c), underpinning the importance of RNA processing proteins in the SCZ ACM.

In CC, up-regulation of nuclear components and down-regulation of secretory organelles, such as endoplasmic reticulum and exosome, were observed in the SCZ cell lysates (Figure 3b). The same secretion-related terms were not significant in the ACM dataset. As for the top 10 CC terms from ACM dataset, ECM and contractile fibre were down-regulated in both modalities, whereas nuclear protein complexes and structures were up-regulated (Figure 3d). Taken together, these results propose a deficit of secretory processes in SCZ astrocytes. However, only the quantity of ECM proteins but not the exosome was altered in the SCZ ACM. The up-regulation of spliceosome echoed the increase of RNA processing proteins in the SCZ ACM.

Finally, in the domain of Molecular Function (MF), all the top 10 terms from cell lysates concerned the binding of DNA, chromatin and histone as well as transcription, yet only the top two of these terms were significant in the ACM dataset: ‘DNA binding’ and ‘chromatin binding’ (Figure S3c). In contrast, ‘RNA binding’ was the topmost dysregulated MF in ACM, followed by the binding of ECM molecules and the binding of chromatin and DNA (Figure S3d).

The strong up-regulation of RNA processing proteins in SCZ ACM (Figure 3a,c,d and Figure S3d) was notable, as nuclear proteins are expected to be the minor contaminants. However, a potential source of them might be extracellular vesicles (EVs) containing nucleic acids and proteins, which have been identified in blood and brain samples of patients with psychiatric disorders, including schizophrenia [48].

To test this hypothesis, we examined the potential involvement of EVs [49,50,51] by visualising the marker proteins thereof on the DEA (control versus SCZ) volcano plots from the two datasets (Figure S3e,f). Although we detected EV marker proteins (e.g., CD63 and CD81), we did not observe a clear trend in regulation for EV marker proteins in lysates and ACM, suggesting that the quantity of EVs was not altered in the SCZ context.

Interestingly, a marker of autophagic EVs, Sequestosome-1 (SQSTM1), was down-regulated at a near-significant level (q-value: 0.017) in the SCZ cell lysates (Figure S3e). The up-regulation of nuclear processes and down-regulation of secretory organelles together with the decrease of the autophagy marker SQSTM1 in SCZ lysates (Figure 3a,b and Figure S3e) point towards the involvement of autophagy-dependent secretion [52] in astrocytic functions in the SCZ context and hence warrant further investigation. Nonetheless, we cannot rule out that the presence of RNA processing proteins in ACM and their relative enrichment in SCZ samples stemmed from artefacts, such as cell debris.

### 3.4. The Effects of SCZ-Associated Donor Background on Astrocytic Proteome

**Figure 4:**
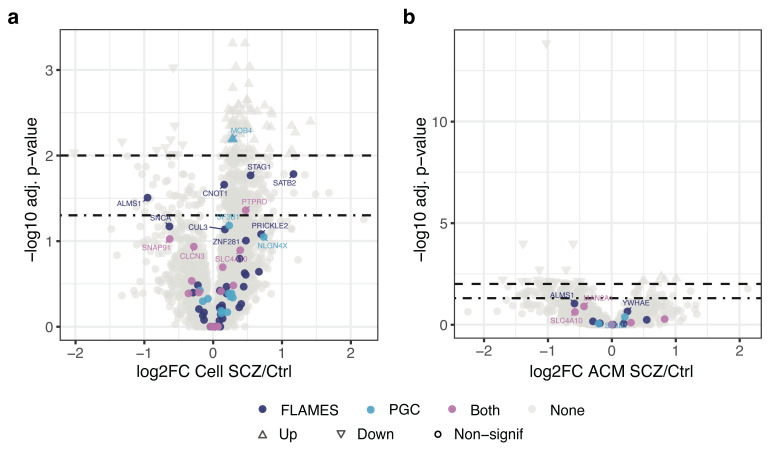
SCZ-associated genes were dysregulated in SCZ astrocytic proteome but not in secretome. (**a**,**b**) Volcano plots from differential expression analyses (DEA) of lysate (**a**) and ACM (**b**) datasets with SCZ-associated genes highlighted. SCZ genes are colour-coded by the publication(s) in which they were reported. Dashed and dashdotted lines denote 1% and 5% FDR cutoff, respectively. Number of proteins tested in DEA and in the SCZ gene lists: lysates—FLAMES = 69, PGC = 30, Both = 24, None = 8090; ACM—FLAMES = 14, PGC = 6, Both = 5, None = 2298.

To evaluate the manifestation of SCZ genetic risk factors in astrocytes in vitro, we intersected differentially expressed proteins with SCZ-associated genes identified by a recent genome-wide association study (GWAS) [53]. Two lists of SCZ genes were tested in our analysis: (1) 120 SCZ genes prioritised by the original paper [53], and (2) 180 effector genes prioritised by the machine learning-based fine-mapping tool, FLAMES [54]. These SCZ genes were subsequently visualised in the DEA (control versus SCZ) volcano plots derived from our cell lysate and ACM datasets (Figure 4a,b). Six GWAS-associated proteins were found dysregulated in the SCZ cell lysates: MOB4 was amongst 88 significant proteins at 1% FDR; SATB2, STAG1, CNOT1, ALMS1 and PTPRD overlapped with 372 significant proteins at 5% FDR (Figure 4a). As for the ACM dataset, there was no overlap between the SCZ genes and 120 significant proteins at 5% FDR (Figure 4b). Notably, five out of six SCZ genes dysregulated in cell lysates were not in the ACM dataset, yet ALMS1 (q-value: 0.092) remained the most affected one amongst the 25 GWAS-associated proteins detected in the ACM samples. The lack of overlap between SCZ genes and the ACM dataset could be due to fewer proteins present in the ACM samples (Figure 1b), or alternatively that SCZ-associated donor background had less impact on the astrocytic secretome.

## 4. Discussion

To study the contribution of astrocytes to the pathophysiology of SCZ from the perspective of intracellular protein expression and secretion, we performed in-depth proteomic analyses of cell lysates and astrocyte-conditioned media (ACM) from iPSC-derived astrocytes of SCZ patients and healthy controls. As expected, fewer proteins were observed in the secretome as compared to the proteome (2884 versus 9037 in the control condition; Figure 1b), and the variation was higher amongst the ACM samples (Figure 1c and Figure S1b), which likely reflects the challenges in consistent media collection. Nevertheless, secretome analysis uniquely captured secreted proteins not readily detected in the cell lysates due to their low abundance (Figure 1d and Figure S1c). GO analyses revealed that the ACM-enriched proteins (as compared to lysates) were associated with ‘extracellular matrix’ (ECM) and ‘extracellular vesicle’ (EV) terms (Figure 1h,i and Figure S1f,g), while the proteins enriched in the lysates (in comparison with ACM) were linked to nuclear and mitochondrial processes (Figure 1f,g and Figure S1d,e), underscoring the complementary strengths of proteome and secretome profiling. Comparison between SCZ and control samples revealed the divergent disease impact on the two modalities. Differentially expressed proteins (DEPs) independently identified from lysate and ACM datasets showed minimal overlap (Figure 2c,d) and the protein effect sizes between the two datasets were weakly correlated (Figure 2e and Figure S2d,e), suggesting that intracellular protein abundance did not reliably predict secretion behaviour under SCZ condition. Importantly, this dissimilarity enabled biologically meaningful classification of dysregulated proteins into lysate-specific, ACM-specific, and co-regulated groups (Figure 2e and Figure S2e).

The ‘ACM-specific’ proteins are particularly informative for secretion-related pathology, as their abundance changed in ACM but remained unchanged in the lysates, suggesting impaired secretion regulation rather than altered synthesis. For example, MFGE8, which is involved in synaptic pruning and neurogenesis [37,38,39,40], and SEMA3C, a key axon guidance cue [41,42], highlight the direct relevance of astrocyte-secreted factors to neural connectivity, supporting a mechanistic link between astrocytic secretion defects and synaptic abnormalities in SCZ. At the pathway level, SCZ effects on proteome and secretome were moderately correlated (Pearson’s r > 0.5; Figure 3e), and both revealed increased nuclear processes and reduced ECM-related components (Figure 3a–d and Figure S3a–d). Yet closer inspection of the strongest gene sets uncovered key differences: up-regulation of the proteins involved in RNA synthesis (Figure 3a) and down-regulation of secretory-organelle signatures (Figure 3b) were only detected in the SCZ lysates, whilst the GO term ‘negative regulation of mRNA metabolic process’ was only significantly up-regulated in the SCZ ACM (Figure 3c). These patterns suggest that intracellular dysregulation of secretion machinery may manifest externally as selective release of nuclear and RNA-processing proteins, although this requires further experimental validation. Together, these results showed that SCZ-associated donor background had distinct impacts on the astrocytic proteome and secretome.

The biological interpretation of the proteins in the ‘lysate-specific’ group could be equivocal, as these proteins were significantly dysregulated in lysates but not in ACM. Several factors may account for this phenomenon: (1) some lysate-specific proteins represented minor contaminants from cell debris in the ACM samples, (2) their secretion was truly comparable between the disease states, and (3) their statistical significance in the ACM dataset was obscured by the high variation between the ACM samples (Figure 1c and Figure S1b). On the other hand, although previous anatomical studies showed no change in glial or astrocyte size in postmortem prefrontal and occipital cortices [44] or hippocampus [55] of SCZ patients, our recent work identified a 12% reduction in cell size of SCZ iPSC-derived astrocytes. We therefore assessed whether the potential unequal cell number per sample biased the lysate measurements. Considering that (1) the total cellular protein mass is theoretically proportional to cell size, (2) equal protein input was used for proteomic analysis, and (3) histone abundance is positively correlated with cell number [46], we reasoned that histone abundance was a good proxy for cell size. The trend of increased histone abundance in SCZ lysates implies slightly higher astrocyte cell counts per equal-protein input (Figure 2g). Importantly, since none of the histone proteins was statistically significant, we concluded that cell size difference was not a systematic confounding factor of significant hits in our proteome-level analysis of SCZ dysregulation (Figure 2a).

Numerous studies have reported the alteration of molecular cargo, such as microRNAs and proteins, in patient-derived brain or blood EVs [56,57,58,59]. For instance, astrocytic miR-223 was found to be increased in the orbitofrontal cortex of SCZ patients, transferred to neurons via exosomes, and inversely correlated with the expression levels of *GRIN2B* and *GRIA2* [56]. Consistent with these observations, the strong up-regulation of RNA processing proteins in the SCZ ACM (Figure 3a,c,d and Figure S3d) suggests that SCZ astrocytes differentially packaged and secreted more EVs containing (micro)RNAs and RNA-binding proteins. Although the abundance of the classic EV markers was unchanged, indicating no alteration of EV quantity (Figure S3e,f), it remains possible that EV cargo composition rather than vesicle number was selectively altered in SCZ. Therefore, to clarify the role of astrocyte-derived EV signalling in SCZ pathophysiology, it is necessary to directly profile the EV-associated RNAs and proteins in future studies. The apparent paradox between reduced GO signatures for secretory organelles (only in lysates; Figure 3b) and unchanged EV marker abundance (in both modalities; Figure S3e,f) could also reflect high variability in ACM (Figure 1c and Figure S1b), frequent media renewal diluting subtle secretion differences, or a selective impact on cargo sorting rather than vesicle biogenesis.

The increase of RNA processing proteins and mitochondrial proteins (e.g., FAHD1 and ACADVL) in the SCZ ACM, together with reduced SQSTM1, a marker for autophagic EV release (q = 0.017; Figure S3e), raised the possibility of enhanced autophagy in the SCZ astrocytes and the concomitant increased release of nuclear and mitochondrial components via autophagic EVs [60,61,62]. However, GO terms related to ‘autophagy’, ‘autophagy of mitochondrion’ and ‘autophagosome’, were not significantly dysregulated in either SCZ astrocytic proteome or secretome. Nevertheless, the perturbation of SQSTM1 raises the possibility that autophagy-mediated secretion [52] contributes to the aberrant release of intracellular materials from SCZ astrocytes, which may compromise astrocytic support for neurons. Alternatively, the enrichment of quintessential intracellular proteins in the SCZ ACM might arise from enhanced vulnerability of SCZ cells.

One of the most biologically compelling findings of our study is the down-regulation of ECM proteins in both astrocytic proteome and secretome. ECM contributes to ~20% of brain volume [63] and is central to tissue structure, synaptic stability, and neural plasticity [64,65]. In the brain, there are three types of ECM: basement membrane (enclosing the pia mater and blood vessels), interstitial matrix (functioning as support and scaffolding), and perineuronal nets (PNNs; mainly responsible for synaptic stabilisation) [64,65]. The reduction of ECM synthesis by the astrocytes could therefore contribute to the known decrease of brain volume in SCZ patients [66,67,68]. Interestingly, numerous studies have reported the decrease of PNN density across multiple brain regions in SCZ patients [69,70,71,72,73,74,75,76]. PNNs are a specialised ECM structure, primarily found around the parvalbumin-containing (PV^+^) GABAergic interneurons, and involved in the modulation of synaptic stabilisation, neural plasticity, and neuronal excitability [65,77,78]; hence, their disruption may underlie excitatory-inhibitory imbalance and cognitive dysfunction in SCZ [79,80]. Importantly, various members of chondroitin sulphate proteoglycans (CSPGs), which is one of the major components of PNNs, are known to be produced by the astrocytes [81]. Thus, the identification of impaired ECM secretion by SCZ astrocytes offers a plausible mechanistic link between astrocyte dysfunction and both macroscale (brain volume) and microscale (PV interneuron deficits) abnormalities in SCZ.

Taken together, our multimodal proteomic study evidences the disruption of intracellular protein homeostasis and secretory processes in the astrocytes harbouring SCZ-associated donor background. In addition, we established an analytical workflow that maximises the complementary strengths of proteome and secretome analyses as well as introduced a histone-based estimation of relative cell size, which will benefit future iPSC-based proteomic studies. Nevertheless, due to the limited sample size (8 Ctrl and 9 SCZ iPSC lines), resulting statistical power, and highly variable nature of the ACM dataset, some truly dysregulated proteins may have remained undetected. On the other hand, further experimental validation of our major findings, including ACM-specific dysregulated proteins MFGE8 and SEMA3C as well as the increased release of RNA-containing EVs by the diseased astrocytes, is needed in order to consolidate their roles in the SCZ pathophysiology. Overall, this work not only reveals the molecular mechanisms whereby the diseased astrocytes could contribute to the synaptic and circuit deficits in SCZ but also highlights astrocyte-mediated extracellular signalling as a promising therapeutic target.

## Data Availability

The mass spectrometry proteomics data have been deposited to the ProteomeXchange Consortium via the PRIDE [82] partner repository with the dataset identifier PXD075039.

## Supplementary Materials

The following supporting information can be downloaded at: https://www.mdpi.com/article/10.3390/cells15121052/s1, Figure S1: Proteomic analysis of iPSC-derived astrocyte cell lysates and ACM; Figure S2: Investigating SCZ-associated dysregulation at the protein level; Figure S3: Investigating SCZ-associated dysregulation at the gene-set level; Table S1: sample metadata of cell-lysate and ACM datasets; Table S2: protein enrichment scores; Table S3: gene-set enrichment in lysates or ACM; Table S4: dea+dd statistics from lysate and ACM datasets; Table S5: gene-set analysis using dea+dd statistics from lysate and ACM datasets.
