# Supplementary material for: Multimodal Proteomics Reveals Dysregulated Secretion and ECM Remodelling in Schizophrenia Patient iPSC-Derived Astrocytes
Source: Cells. 2026 Jun 9;15(12):1052. doi: 10.3390/cells15121052 (PMC13297010; doi:10.3390/cells15121052)
Supplement: Supplementary file 1 [file cells-15-01052-s001.zip › Li at al_supporting_information.pdf]

Multimodal Proteomics reveals dysregulated secretion and ECM remodelling in schizophrenia patient iPSC-derived astrocytes

Wei-Ping Li[1,2,#], Karen E. Laupman[1,#], Stephanie D. Beekhuis-Hoekstra[1], Evangelia Thanou[2], Remco V. Klaassen[2], Patrick F. Sullivan[4,5], Danielle Posthuma[1,3], August B. Smit[2], Frank Koopmans[2,$], Vivi M. Heine[1,3,$,*]

1. Department of Complex Trait Genetics, Center for Neurogenomics and Cognitive Research, Vrije Universiteit Amsterdam, Amsterdam, The Netherlands.
2. Department of Molecular and Cellular Neurobiology, Center for Neurogenomics and Cognitive Research, Vrije Universiteit Amsterdam, Amsterdam, The Netherlands.
3. Department of Child and Adolescent Psychiatry, Amsterdam Neuroscience, Vrije Universiteit Medical Center, Amsterdam, The Netherlands.
4. Department of Medical Epidemiology and Biostatistics, Karolinska Institutet, Stockholm, Sweden.
5. Department of Genetics, University of North Carolina, Chapel Hill, NC, 27599-7264, USA.

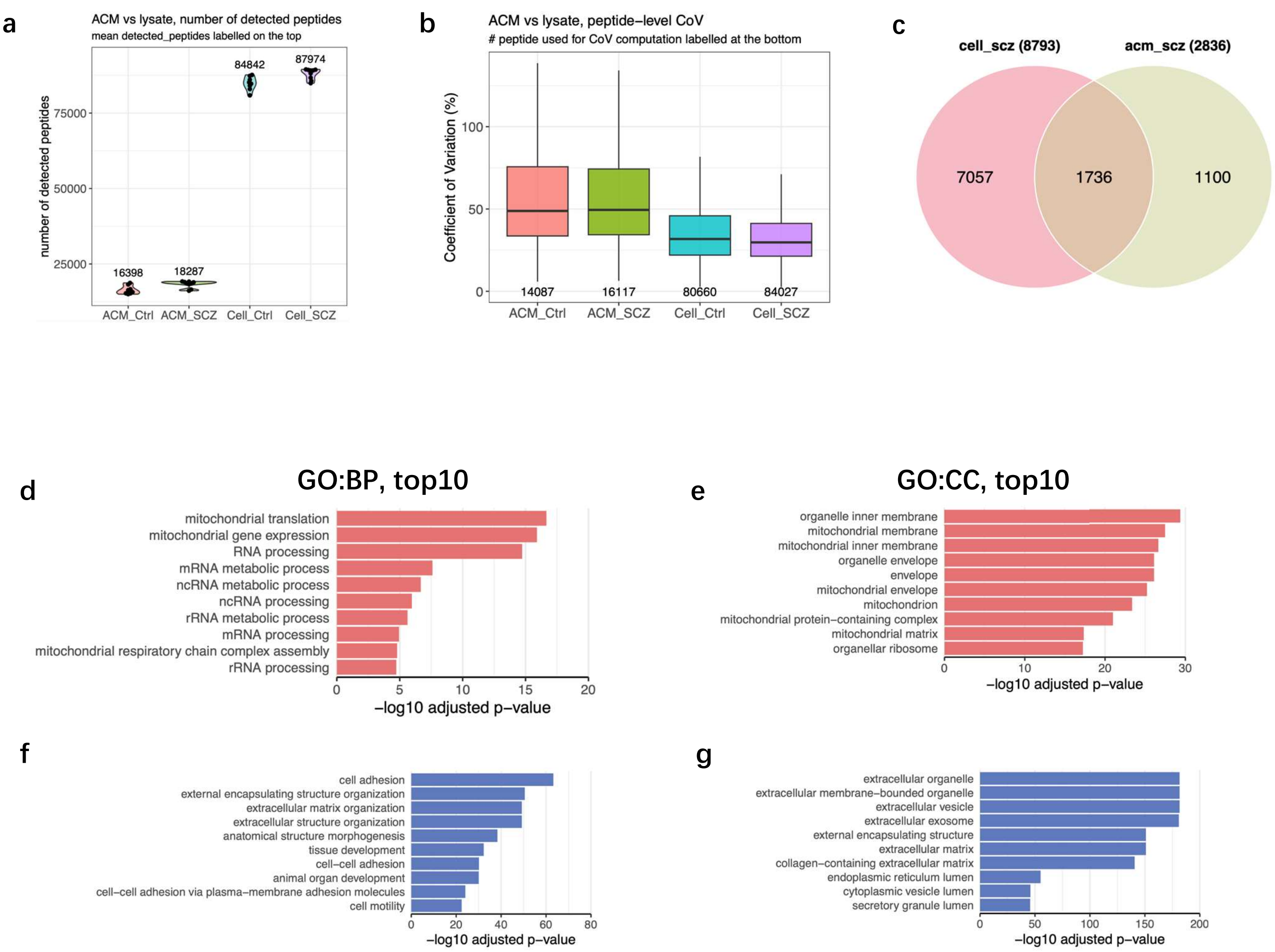

Figure S1. Proteomic analysis of iPSC-derived astrocyte cell lysates and ACM.

a) Number of detected peptides in each experimental condition. The mean value is shown for each sample group. **b**) Distributions of peptide (abundance) variation in each experimental condition. Only peptides identified in at least 3 replicates (peptide numbers are displayed at the bottom) were included in the computation of CoV. **c**) Overlap of proteins consistently detected (≥75% samples/group) in SCZ lysates and ACM. **d,e**) Top 10 biological process (BP) and cellular component (CC) terms that were enriched in SCZ lysates as compared to ACM (full results in Table S3: SCZ_enriched in lysates). **f,g**) Analogous to panels d,e yet showing GO terms enriched in SCZ ACM as compared to lysates (full results in Table S3: SCZ_enriched in ACM).

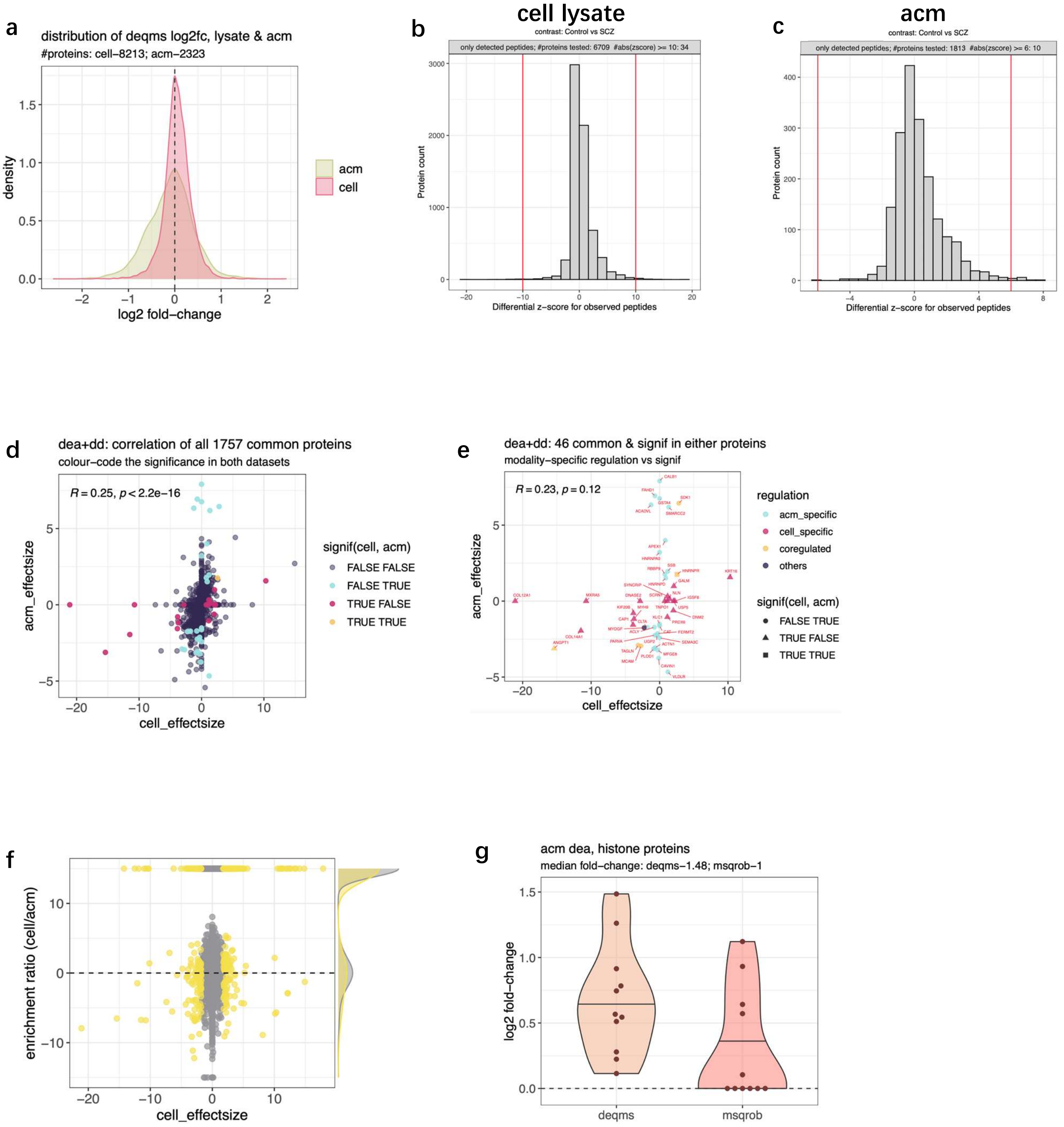

Figure S2. Investigating SCZ-associated dysregulation at the protein level.

a) Distributions of protein log_2 fold changes in SCZ lysates and ACM estimated by DEqMS statistical model. **b,c**) Histograms showing the number of proteins (y-axis) with the corresponding control *vs* SCZ differential detection z-scores (x-axis) in lysate (b) and ACM (c) samples. **d**) Comparison of protein control *vs* SCZ effect sizes between lysates (x-axis) and ACM (y-axis) for the subset of 1,757 proteins that are available for statistical analysis in both statistical contrasts. **e**) Fig. 2e with gene symbols labelled. **f**) Relationship between the

control *vs* SCZ effect sizes in lysates (x-axis) and enrichment ratios (y-axis; control ratios in Fig. 1e). The top 10% proteins with strongest (absolute) effect sizes in lysates are highlighted in yellow. **g**) Histone proteins are enriched in SCZ ACM as compared to controls, implying the relative vulnerability of SCZ cells. Log_2 fold changes for each protein are shown (y-axis) for both the MSqRob and DEqMS statistical models (x-axis).

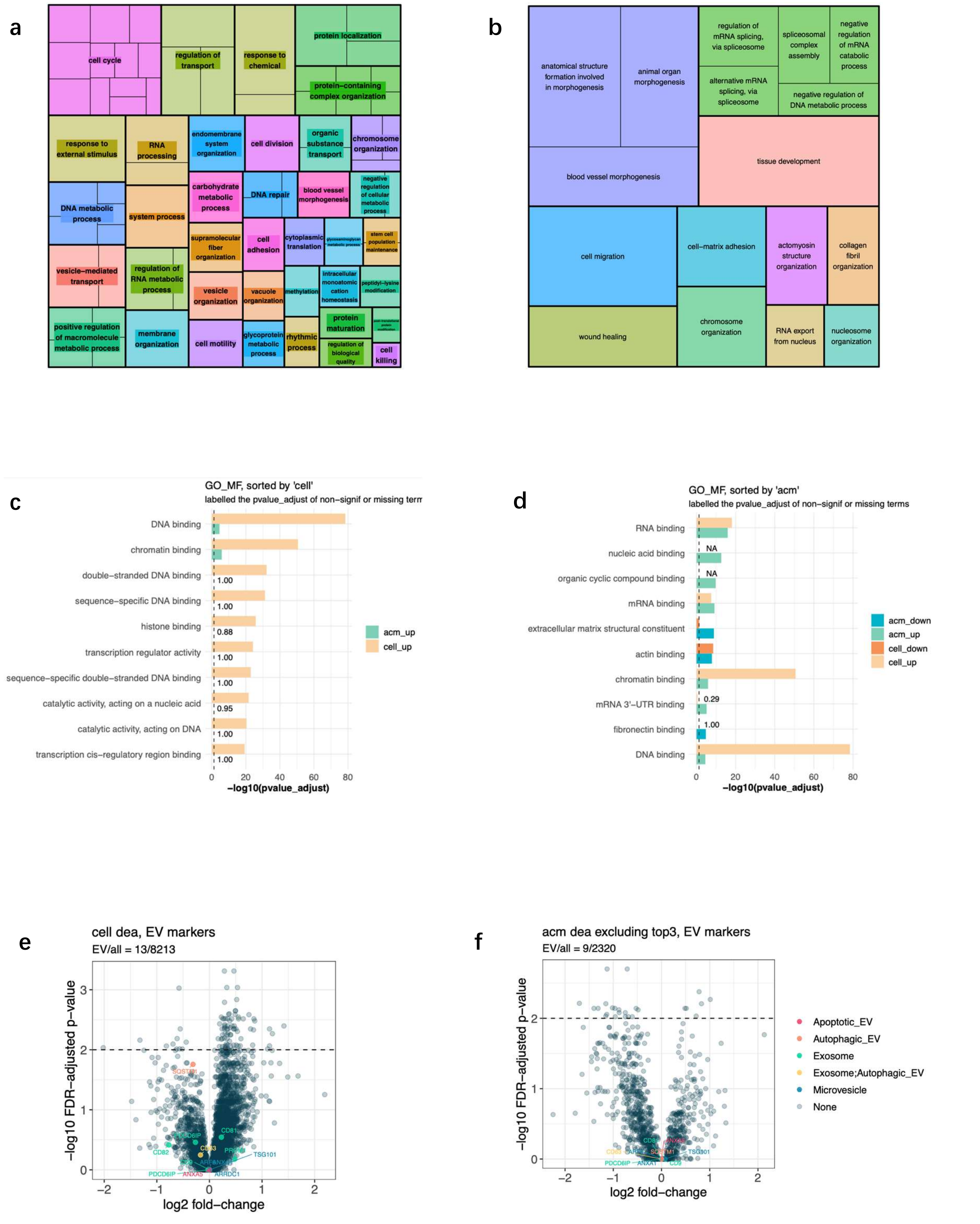

Figure S3. Investigating SCZ-associated dysregulation at the gene-set level.

a,b) Treemap plots summarising significant biological process (BP) terms identified from lysate (a) and ACM (b) datasets. For visual clarity, only the group names are shown in panel a. **c,d**) Top 10 molecular function (MF) terms identified by gene set analyses of lysate (c) and ACM (d) samples. The dataset from which the significance (adjusted p-value) was derived is

colour-coded in teal (ACM) and orange (lysates). Lighter and darker colours denote up- and down-regulation, respectively. **e,f**) Volcano plots from differential expression analyses (DEA) of lysate (e) and ACM (f) datasets with marker proteins of different types of extracellular vesicles (EVs) highlighted. For visual clarity, the top 3 significant proteins are excluded from panel f.
